# Maternal and fetal outcomes of pregnant women with bacterial vaginosis

**DOI:** 10.3389/fsurg.2023.1084867

**Published:** 2023-02-13

**Authors:** Beng Kwang Ng, Joo Ngor Chuah, Fook Choe Cheah, Nor Azlin Mohamed Ismail, Geok Chin Tan, Kon Ken Wong, Pei Shan Lim

**Affiliations:** ^1^ Department of Obstetrics and Gynaecology, Faculty of Medicine, Universiti Kebangsaan Malaysia, Kuala Lumpur, Malaysia; ^2^ Department of Paediatrics, Faculty of Medicine, Universiti Kebangsaan Malaysia, Kuala Lumpur, Malaysia; ^3^ Department of Pathology, Faculty of Medicine, Universiti Kebangsaan Malaysia, Kuala Lumpur, Malaysia; ^4^ Department of Medical Microbiology and Immunology, Faculty of Medicine, Universiti Kebangsaan Malaysia, Kuala Lumpur, Malaysia

**Keywords:** preterm birth, preterm labour, BV® blue, neonatal intensive care unit, Gardnerella vaginalis, vaginal discharge, rupture of membrane, histologic chorioamnionitis

## Abstract

**Background:**

Bacterial vaginosis (BV) is a common infection in women of reproductive age group because of vaginal dysbiosis. The impact of BV during pregnancy is still not well defined. The objective of this study is to assess the maternal-fetal outcome in women with BV.

**Materials and Methods:**

A prospective cohort study over one-year duration was conducted from December, 2014 until December, 2015, involving 237 women who presented with abnormal vaginal discharge, preterm labour or preterm prelabour rupture of membrane between 22- and 34-weeks period of gestation. Vaginal swabs were sent for culture and sensitivity, BV® Blue testing and PCR for Gardnerella vaginalis (GV).

**Results:**

BV was diagnosed in 24/237 (10.1%) cases. The median gestational age was 31.6 weeks. GV was isolated from 16 out of 24 (66.7%) in the BV positive group. There was a significantly higher preterm birth rate, below 34 weeks (22.7% vs. 6.2%, *p* = 0.019) in women with BV. There was no statistically significant difference in maternal outcome such as clinical chorioamnionitis or endometritis. However, placental pathology revealed more than half (55.6%) of women with BV had histologic chorioamnionitis. Neonatal morbidity was significantly higher with exposure to BV, with a lower median birth weight, higher rate of neonatal intensive care unit admission (41.7% vs. 19.0%, *p* = 0.010), increased intubation for respiratory support (29.2% vs. 7.6%, *p* = 0.004) and respiratory distress syndrome (33.3% vs. 9.0%, *p* = 0.002).

**Conclusion:**

More research is needed to formulate guidelines for prevention, early detection and treatment of BV during pregnancy to reduce intrauterine inflammation and the associated adverse fetal outcomes.

## Introduction

Bacterial vaginosis (BV) is an imbalance of normal genital tract microbiota, where depletion of hydrogen-peroxide producing lactobacilli is replaced by *Gardnerella vaginalis* (GV), *Mobiluncus, Bacteroides spp*, and *Mycoplasma hominis* (1, 2). Gardner and Duke reported a syndrome called non-specific vaginitis in the 1950s, which was re-named bacterial vaginosis. Without using anaerobic culture techniques, the microaerophilic microorganism Haemophilus (*Corynebacterium, now renamed Gardnerella*) vaginalis was said to be the sole etiology of BV (3). With today's improved culture and diagnostic technique, BV consists mainly of anaerobic bacteria in the mixed flora. These obligate anaerobic bacteria appear side by side with *Gardnerella vaginalis and Mycoplasma hominis* (3).

BV is a common disorder in women of childbearing age. Women may have classical symptoms of grey, homogenous malodorous discharge but up to 80% of them were asymptomatic (4). It affects 6.4 to 16% of pregnant women (5–9) and has been linked to several obstetrics complications including preterm birth, preterm premature rupture of membrane, intra-amniotic infection, postpartum endometritis, as well as neonatal complications i.e., respiratory distress syndrome (RDS) and neonatal intensive care unit (NICU) admission (10–12). Even so, reports of associated complications are still variable and not consistent, depending on the origin of the studies.

The diagnosis of BV remains difficult and controversial. Amsel's criteria include the homogenous greyish white appearance of vaginal discharge, presence of clue cells on a wet mount, vaginal pH of more than 4.5 and a positive whiff test. The diagnosis of BV requires 3 out of 4 criteria (13). On the other hand, Nugent's scoring system (0 to 10) was described as a weighted combination of the following morphotypes: *Lactobacilli sp*, *Gardnerella vaginalis* or *Bacteroides sp*, and curved gram variable rods (14). In this method, a score of 0–3 represents normal flora, 4–6 intermediate, and 7–10 as BV. The sensitivity and specificity of Amsel criteria were 51.2–88.3% and 92%–98% respectively (7, 15); whereas Nugent's score was 46%–89% and 83%–95% respectively (15, 16). However, both methods required trained personnel for slide preparation and result interpretation which could be a major drawback.

Recently, a bedside rapid test to diagnose BV has gained much popularity. The BV® Blue test is a chromogenic diagnostic test based on the presence of elevated sialidase enzyme in vaginal fluid samples that were produced by organisms causing BV (17). The sensitivity and specificity reported were 88%–100% and 95–98.3% as compared to Nugent's method (17–19). Whereas, the positive predictive value (PPV) and negative predictive value (NPV) were 91.7–94.4% and 97.8%–100% (17, 18). Thus BV® Blue test was chosen to diagnose BV in this study.

With the advancement in cultivation-independent methods like Polymerase Chain Reaction (PCR), the presence of newly diagnosed vaginal species such as *Fannyhessea vaginae* (formerly known as *Atopobium vaginae*) and three bacterial species in the *Clostridiales* order that were highly specific for BV were revealed (20). A study using semi-quantitative multiplex PCR assay by Kusters et al. showed the presence of GV in 96% of women with a Nugent score of 7–10 and GV only present in 27% if Nugent score was 1–3 (21). By using this technique, the BV-PCR displayed a sensitivity of 92% and specificity of 96% with a PPV and NPV of 94% and 95% respectively (21). Another study by Rumyantseva et al. evaluated the diagnostic value of Nugent score, wet mount microscopy and PCR test and showed that agreement among the three methods was 73.5% (72 out of 98 samples) (22). PCR quantifies Deoxyribonucleic Acid (DNA) rather than the viable organism. This is a potential advantage as it can detect the organism in archived genital tract samples that were collected under a condition that were not optimized for organism viability. PCR is also highly sensitive and able to pick up a very low number of bacteria.

Isolation of GV in BV may be of clinical and therapeutic importance. A high bacterial load of GV was found to be associated with preterm birth, with a hazard ratio of 3.9 (23). Molecularly, GV was reported to be the major component that was responsible for 90% of bacterial biofilm on vaginal epithelium (24). GV biofilms also exhibited higher tolerance to hydrogen peroxide, initiate BV establishment, facilitate the growth of other BV-associated anaerobes and resist repeated intravaginal antiseptic treatment (25, 26).

In Malaysia, there has been a paucity of data concerning maternal and fetal outcome in women with BV. The objectives of this study were to assess the feto-maternal outcome in women diagnosed to have BV. The prevalence of GV in BV-positive women was assessed as well.

## Materials and methods

### Study design

Pregnant women who presented with abnormal vaginal discharge, preterm pre-labour rupture of membrane (PPROM) or preterm labour, gestational age from 22 weeks to 34 weeks, singleton pregnancy, and those who consented to the study were recruited. Abnormal vaginal discharge was defined as a change in colour (such as grey, green or yellow, or blood-stained), copious amount, or odour, associated with itchiness or soreness. Preterm labour was defined as having regular contractions of at least 2 in 10 min with cervical effacement and cervical os dilatation. Pregnant women with the following criteria were excluded: obstetric complications that can be confounding factors for preterm delivery such as pre-existing medical disorders e.g., diabetes, hypertension, cardiac and renal disease, and all complicated pregnancies (antepartum hemorrhage, fetal anomaly, multiple gestation, intrauterine growth restriction, or polyhydramnios. Oligohydramnios caused by IUGR or fetal anomalies were excluded. However, oligohydramnios following PPROM during enrolment was not our exclusion criteria). Women who had cervical incompetence, uterine or cervical anomaly, fetal death, and history of recent douching, or sexual intercourse pre-testing, as well as recent use of systemic or vaginal antimicrobial therapy either as suppository drugs or spray within the preceding 72 h were excluded from the study.

### Procedure

The study was conducted according to the guidelines of the Declaration of Helsinki, and approved by the Medical Research and Ethics Committee of Universiti Kebangsaan Malaysia (FF-2015-037). All eligible pregnant women were informed about the study and they were provided with a patient information leaflet. Written consent was obtained. A detailed history, physical examination, and sterile speculum examination were performed either in the antenatal clinic or patient admission centre of the Department of Obstetrics and Gynaecology, Universiti Kebangsaan Malaysia Medical Centre. All the details were recorded on a proforma.

Vaginal fluid was collected during speculum examination in the following manner. Women were positioned in the dorsal position. A Cusco speculum was inserted without any lubricant. Characteristics of the vaginal discharge were noted. Three samples were then obtained from the posterior vaginal fornix using sterile cotton-tipped swabs. The first swab was sent for culture and sensitivity to look for any organism that might cause the vaginal discharge such as *Candida spp* or *Group B Streptococcus*. The second sample was obtained for BV® Blue testing to diagnose BV.

For the BV® Blue test, the swab was immersed into the BV® Blue Testing Vessel that contained a chromogenic substrate of bacterial sialidase at room temperature (24–32 °C). The vessel was left standing for 10 min. The test vessel was checked to ensure it contained only colourless fluid without sediment. Subsequently, one drop of BV® Blue Developer Solution was added into the testing vessel and swirled. Then, the result was read immediately. A positive result was interpreted as a change of colour to blue or green while yellow was a negative result. If the result was not blue/green or yellow, then the test was repeated. For those with BV® Blue tested positive, the third swab would be sent to the laboratory for detection of GV using the PCR method. For those who tested negative, the third swab for GV was discarded. To reduce interpersonal data interpretation errors, these tests were performed by a single operator.

### PCR setup

GV was selected in this study as it was thought to be the main contributing bacteria in bacterial vaginosis. Isolation of its DNA from the vaginal swab was performed using InnuPREP DNA Mini Kit based on the extraction protocol as advised by the manufacturer. The extraction procedure involved a lysing step, followed by binding of genomic DNA on a Spin Filter surface, washing of the bound DNA, and eluting of the DNA. The presence of GV was established by qualitative PCR. The amplifications were carried out in an automated Gene Amp PCR System 9,700 (Perkin-Elmer Cetus, CT, USA) using MyTaq™ HS Mix as specified by the manufacturer. Forward and reverse primers were used (GVF: TTCGATTCTGGCTCAGG and GVR: CCATCCCAAAAGGGTTAGGC) at the concentration of 0.25 *μ*M. Reactions were performed in a final volume of 25 μl. PCR conditions consisted of a heat-activated Taq polymerase of 95 °C for 60 s followed by 35 cycles of amplification. Each amplification cycle consisted of a denaturation step at 95 °C for 15 s, an annealing step at 55 °C for 15 s and finally extension step at 72 °C for 10 s. The PCR product is detected by 1.2% (w/v) agarose gel electrophoresis with 1X Tris/Borate/EDTA (TBE) buffer. The GV (ATCC 14108) was used as a positive control along with a “No Template Control” and were amplified together with the samples.

All patients with a positive BV® blue test were treated with Dequalinium Chloride vaginal pessary (Fluomizin® Medinova, DKSH) 1 tablet daily for 6 days. Other than Dequalinium chloride vaginal pessary, patients with preterm labour or PPROM were also managed per standard hospital protocol. All patients were followed up till delivery and post-partum. The outcomes of pregnancy were recorded for both the mother and baby. Upon delivery, a small portion of the placenta tissue was sent for histopathology examination for evidence of chorioamnionitis. The neonatal outcomes such as the need for intubation, antibiotic administration, days of admission to NICU, presence of respiratory distress syndrome, baby's cord pH, Apgar score, and neonatal death were recorded. Patients were called *via* phone at six weeks postpartum by the investigator to enquire about any postpartum complications such as postpartum endometritis or wound breakdown. Questions included a history of fever and persistent lochia which was excessive, purulent, or malodorous to suggest endometritis or history of discharge or pus from the wound, redness, and pain, suggestive of wound breakdown. If they have such a complaint, they were asked to present to the patient admission centre immediately.

### Data analysis

SPSS (Statistical Package for Social Science) version 23 was used for data analysis. Participants' profiles were presented descriptively in terms of frequency and percentage, mean and standard deviation (for normally distributed data), or median and interquartile range (for non-normally distributed data). Non-parametric statistical tests (Mann-Whitney test) were used for variables such as maternal age, monthly income, gestational age during recruitment, gestational age during delivery, the interval between recruitment and delivery (Tables 1,2) as well as birth weight and cord pH (Table 3). The chi square test was used to compare two categorical data in Tables 1–3. A significant level was set at *p* < 0.05.

**Table 1:** Patient socio-demographic characteristics.

	All (*n* = 237)	BV positive (*n* = 24)	BV negative (*n* = 213)	*p*-value
Maternal age (years)	30.0 (27.0,33.0)	29.0 (26.0, 32.0)	30.0 (27.0, 33.0)	*p* = 0.321
Marital status, *n* (%)				
• Married	233 (98.3)	24 (100.0)	209 (98.1)	*p* = 0.498
• Single	3 (1.7)	0 (0.0)	4 (1.9)	
Ethnicity, *n* (%)				
• Malay	161 (67.9)	16 (66.7)	145 (68.1)	*p* = 0.670
• Chinese	55 (23.2)	7 (29.2)	48 (22.5)	
• Indian	10 (4.2)	0 (0.0)	10 (4.7)	
• Others	11 (4.6)	1 (4.2)	10 (4.7)	
Educational level, *n* (%)				
• Primary	8 (3.4)	0 (0.0)	8 (3.8)	*p* = 0.409
• Secondary	104 (43.9)	13 (54.2)	91 (42.7)	
• Tertiary	125 (52.7)	11 (45.8)	114 (53.5)	
Monthly income (RM)	3,500 (3,000, 5,000)	4,000 (2,575, 5,000)	3,500 (3,000, 4,950)	*p* = 0.676
Nulliparous, *n* (%)	101 (42.6)	13 (54.2)	88 (41.3)	*p* = 0.227

Data expressed in median (Quartile) unless specified.

**Table 2:** Patient clinical characteristics.

	All (237)	BV positive (24)	BV negative (213)	*p*-value
Presenting complaint, *n* (%)				
• Abnormal vaginal discharge	173 (73.0)	16 (66.7)	157 (73.7)	*p* = 0.742
• Preterm labour	46 (19.4)	6 (25.0)	40 (18.8)	
• PPROM	18 (7.6)	2 (8.3)	16 (7.5)	
Gestational age at recruitment, weeks	31.6 (28.5, 33.1)	32.1 (27.7, 32.9)	31.1 (28.5,33.1)	*p* = 0.768
• Abnormal vaginal discharge	30.9 (28.1,33.0)	32.2 (30.0,32.9)	30.7 (28.0,33.0)	*p* = 0.330
• Preterm labour	32.1(30.0,33.6)	32.0 (26.3,33.2)	32.1 (30.0,33.6)	*p* = 0.493
• PPROM	32.8 (28.2,33.4)	29.1 (24.9,-)	32.8 (29.2,33.4)	*p* = 0.526
Gestational age at delivery, weeks	38.0 (36.4, 39.1)	37.3 (34.0, 38.3)	38.1 (36.7, 39.3)	*p* = 0.010
• Abnormal vaginal discharge	38.3 (37.4,39.3)	38.0 (36.0, 38.9)	38.4 (37.6,39.6)	*p* = 0.077
• Preterm labour	37.0 (34.0,38.4)	33.8 (32.3, 37.6)	37.0 (34.7, 38.9)	*p* = 0.108
• PPROM	34.8 (33.9,37.0)	34.2 (33.9, -)	35.2 (33.9,37.0)	*p* = 0.439
Interval between recruitment and delivery, weeks	6.9 (4.5,10.0)	4.8 (1.5,8.9)	7.2 (4.7,10.0)	*p* = 0.026
• Abnormal vaginal discharge	7.8 (5.2,10.5)	5.5 (2.9,9.2)	8.0 (5.4,10.7)	*p* = 0.097
• Preterm labour	4.6 (0.6,7.2)	0.6 (0.0, 11.2)	4.9 (2.8,7.0)	*p* = 0.369
• PPROM	3.3 (1.0,6.0)	5.2 (1.3,-)	3.3 (1.0,5.6)	*p* = 0.673
Preterm birth <34 weeks, n(%)	17 (7.9)	5 (22.7)	12 (6.2)	*p* = 0.019
• Abnormal vaginal discharge	2 (0.9)	1 (4.5)	1(0.5)	*p* = 0.177
• Preterm labour	10 (4.7)	3 (13.6)	7 (3.6)	*p* = 0.107
• PPROM	5 (2.3)	1 (4.5)	4 (2.1)	*p* = 0.490
Mode of delivery, *n* (%)				
• Vaginal delivery	177 (74.7)	16 (66.7)	161 (75.6)	*p* = 0.624
• Caesarean Section	60 (25.3)	8 (33.3)	52 (24.4)	
Concurrent infection, *n* (%)	101 (42.6)	12 (50.0)	89 (41.8)	*p* = 0.367

All parameters are expressed in median (interquartile range) unless specified. PPROM, Preterm prelabour rupture of membrane.

^a^ Total = 17/215, excluding 22 cases who were recruited after 33 weeks; BV positive, *n* = 5/22; BV negative, *n* = 12/193.

**Table 3:** Maternal-fetal outcome according to bacterial vaginosis status.

	All (*n* = 237)	BV positive (*n* = 24)	BV negative (*n* = 213)	*p*-value
Primary PPH, *n* (%)	4 (1.7)	0 (0.0)	4 (1.9)	*p* = 0.498
Endometritis, *n* (%)	3 (1.3)	1 (4.2)	2 (0.9)	*p* = 0.180
Wound breakdown, *n* (%)	2 (0.8)	1 (4.2)	1 (0.5)	*p* = 0.061
Birth weight (grams)	2,930 (2,510, 3,195)	2,450 (2,150, 2,957)	2,950(2,600, 3,200)	*p* = 0.007
Apgar score ≤7 at 5th minute	13 (5.6)	3 (12.5)	10 (4.8)	*p* = 0.117
Cord pH	7.324 (7.265, 7.367)	7.256 (7.227, 7.343)	7.330 (7.275, 7.369)	*p* = 0.004
NICU admission, *n* (%)	50 (21.4)	10 (41.7)	40 (19.0)	*p* = 0.010
Baby intubated, *n* (%)	23 (9.8)	7 (29.2)	16 (7.6)	*p* = 0.004
RDS, *n* (%)	27 (11.5)	8 (33.3)	19 (9.0)	*p* = 0.002
Perinatal death, *n* (%)	5 (2.1)	0 (0.0)	5 (2.3)	*p* = 0.448

## Results

A total of 251 pregnant women were eligible and consented to the study. However, 14 were excluded as eight of them were lost to follow-up after being discharged from the ward and the other four had their deliveries at other hospitals, and the delivery information was unable to be traced. Two women refused to give consent for placental HPE to be conducted in this study. Thus, we had a response rate of 94.4% i.e., 237 pregnant women were available for final data analysis. The demographic data were shown in Table 1. The median age for the study sample was 30.0 years (27.0, 33.0). It was comparable in both the positive group (BV positive) and the control group (BV negative). Almost all of the pregnant women were married (98.3%) and the ethnic distribution was similar to the ethnic distribution in the country. The majority were Malay (67.9%), followed by Chinese (23.2%), Indian (4.2%), and others (4.6%). More than half of the pregnant women were multiparous (58.4%) with a median monthly income of 3,500 Malaysian Ringgit (approximately 750 US dollars). The demographic data were similar between the positive and the control group (Table 1).

Further analysis of patients' clinical characteristics showed that the majority of them presented with abnormal vaginal discharge (73.0%), followed by preterm labour (19.4%) and least commonly, PPROM (7.6%). This was not different statistically between the positive and control group (*p* = 0.772). The median gestational age at recruitment was 31.6 weeks and it was comparable in both the positive and control group (32.1 vs. 31.1, *p* = 0.768). However, patients in the BV positive group had a significantly higher percentage of preterm birth, below 34 weeks gestation (22.7% vs. 6.2%, *p* = 0.019) and lower gestational age upon delivery (37.3 vs. 38.1 weeks, *p* = 0.010) as compared to the negative control group. Thus, the interval between patient recruitment and delivery was also noted to be significantly shorter in the positive group as compared to the control group (4.8 vs. 7.2 weeks, *p* = 0.026). In analysing the three subgroups separately, it is noteworthy that the gestational ages at recruitment were similar, however, gestational age at delivery was significantly lower in the BV positive group, being shortest in the preterm labour group, median 33.8 weeks vs. 37.0 weeks in the BV negative group. This is not statistically significant when looking at each subgroup separately, likely because the sample size is small. Collectively, the BV-positive subgroup also showed a significantly shorter average interval (4.8 weeks) from presentation and recruitment to delivery as opposed to the BV-negative group (7.2 weeks) (Table 2). There was no statistically significant difference in the mode of delivery (vaginal delivery vs. caesarean section) between the two groups (*p* = 0.624). One in two patients with BV positive also had concurrent vaginal infections such as candidiasis and group B streptococcus infection but this was not significantly different from the BV negative group (Table 2).

There was no statistically significant difference in any maternal outcome such as primary postpartum haemorrhage (PPH), endometritis and wound breakdown between the positive and control groups (*p* = 0.498, *p* = 0.180 and *p* = 0.061 respectively). Two patients (one in each group) had perineal wound breakdown after vaginal delivery (Table 3). There was no overt/clinical chorioamnionitis in both groups. However, babies born to mothers with BV had significantly lower median birth weight (2,450 grams vs. 2,950 grams, *p* = 0.007). These babies were also noted to have a lower median cord pH (7.256 vs. 7.330, *p* = 0.004), which was of doubtful clinical relevance as it was still within the normal range. Of note, babies born to mothers with BV had more than double the NICU admission rate (41.7% vs. 19.0%, *p* = 0.010), almost four-fold higher rate of endotracheal intubation (29.2%% vs. 7.6%, *p* = 0.004) and RDS (33.3% vs. 9.0%, *p* = 0.002). There was no perinatal death in the BV positive group as compared to the control group (*n* = 5) (Table 3) The placental HPE showed evidence of acute chorioamnionitis up to 55.6% in the BV positive group. GV was positive in 16 out of 24 BV positive patients.

## Discussion

The prevalence of BV in this study was 10.1% (diagnosis by BV® Blue test). The reported prevalence in the literature was widely different based on different populations of study samples, diagnostic tools used, and whether the studies were done in a community setting or academic medical centre (27).

This study was consistent with a cohort study done by Purmar et al. among 1,006 pregnant women between 16 and 28 weeks gestation, in which the prevalence of BV was reported to be 11.5% (by Nugent's criteria) (6). Larsson et al. (8) reported similar findings in their reviews. However, the prevalence of this study was lower than the review by Svare et al. among 3,540 pregnant women at University Hospital Denmark, in which BV was detected in 16% of their subjects (9). Our study was conducted on pregnant women who presented with vaginal discharge, preterm labour and PPROM. We would expect our finding of BV to be higher as compared to others that studied asymptomatic pregnant women, which were considered a low-risk population. Looking at the prevalence of BV among 152 women with preterm labour, Laxmi et al. reported the detection of BV up to 24.3% in their subjects (12). Another study by Thanavuth et al. reported that the prevalence of BV was higher in women with preterm labour as compared to women presented with preterm contraction only (25.8% vs. 14.1%) (28). In our study, the prevalence of BV in patients who presented with preterm birth was 20.9%.

Previous studies had looked into risk factors for antenatal BV. Larsson et al. reported that the prevalence of BV was significantly higher in women who smoke and in the younger age group, but not in those with a history of previous preterm delivery (8). Whereas, Kirakoya et al. identified Herpes Simplex Virus type-2 infection as the only factor associated with an increased risk of BV (5). Pastole et al. concluded that 6 predictors i.e., vaginal pH > 4.5, black race, condom use during pregnancy, antenatal BV earlier in the index pregnancy, absence of sperm on smear, and no history of sexually transmitted disease could be used to predict the risk of antenatal BV. This scoring system had a sensitivity and specificity of 77% (4). In this study, we were unable to demonstrate any significant association between maternal age, marital status, educational level, and parity concerning the risk of BV.

Even though most of the women with BV infection were asymptomatic (4), efforts should be made to identify and diagnose this infection when pregnant women presented to health care providers as it was associated with adverse fetal outcomes. Most of our patients with BV positive presented with abnormal vaginal discharge instead of preterm labour or PPROM. Thus, a simple and inexpensive bedside rapid test to diagnose BV should be made available in all settings where possible, allowing rapid commencement of treatment. We used the BV® Blue test to diagnose BV in this study. The sensitivity and specificity reported were 88%–100% and 95–98.3% as compared to Nugent's method (17–19), and the positive predictive value (PPV) and negative predictive value (NPV) were 91.7–94.4% and 97.8%–100% (17, 18).

In this study, we demonstrated that BV-positive women had a statistically significant higher rate of moderate preterm birth (below 34 weeks gestation), up to almost four-fold, and delivered at an earlier gestational age despite being given treatment. In a sub-analysis of women with preterm labour only, BV-positive women delivered at the median gestational age of about 34.0 weeks as compared to 37 weeks in those who were BV-negative. This was consistent with a meta-analysis by Leitich et al. that included eight studies with 20,232 patients. Bacterial vaginosis was associated with a 2-fold increased risk of preterm delivery with an odds ratio of 2.9 (2). A similar finding was reported by Purwar et al. in which the incidence of preterm labour and PPROM was significantly higher in BV positive women as compared to BV negative women (*p* = 0.001). Our study also showed that in BV positive women presenting with vaginal discharge alone, the average gestational age at delivery was 38 weeks with a shorter average interval to delivery of 5.5 weeks. This was not statistically significant. Larger population sampling from international multicentre trials is needed to confirm this important observation.

The overall preterm delivery rate in this study was as high as 24.2% (gestational age range from 23.9 to 36.9 weeks) with the prevalence of BV at 10.1%. This was contrary to the study by Svare et al. in which the prevalence of BV was 16% in 3,540 women and the preterm delivery rate was only 5.2%. In their review, BV had a statistically significant association with preterm delivery, low birth weight infant and clinical chorioamnionitis (9). On the other hand, Donders et al. found that the presence of BV was associated with a 2.4-fold increased risk of preterm delivery (10). This was consistent with the study by Guaschino et al. in which the presence of BV before 16-week gestation had a 2-fold increased risk of preterm birth (11). In our study, 50% (9 out of 18) of those with BV positive had a preterm birth, whereas only 21% of those with BV negative had a preterm birth.

There was no statistically significant association between adverse maternal outcomes and bacterial vaginosis in this study. The rate of primary PPH between the two groups was not different. The rate of endometritis and wound breakdown were not significantly higher in BV-positive women, consistent with the study by Larsson et al. (8). Conversely, Watts et al. reported that BV was a risk factor for post-caesarean endometritis (29). However, our numbers for adverse maternal outcomes were too small to draw any solid conclusion.

BV was a risk factor for increased neonatal morbidity as demonstrated by previous studies (12, 29). A study by Laxmi et al. reported that admission to NICU, NICU stays of more than 2 days, the need for intermittent positive pressure ventilation and RDS were higher in infants born to women with BV infections (12). However, there was no difference in mean birth weight, Apgar score at 5 min, or risk of neonatal sepsis or perinatal mortality (12). Another study by Subtil et al. showed a significant difference in Apgar score at 5 min but not the risk of NICU admission or perinatal death (30). In our study, we demonstrated that babies born to BV-positive women had a significantly higher rate of admission to NICU, need for intubation, and RDS. The length of stay in the NICU ranged from 2 to 7 days. The placental HPE showed evidence of acute chorioamnionitis in up to 55.6% of the BV positive cases.

A recent Cochrane review by Sangkomhang et al. revealed that antenatal lower genital tract infection screening and treatment reduced the rate of preterm birth significantly and is cost-saving (31). Another Cochrane review of 21 good quality trials by Brocklehurst et al. demonstrated that antibiotic treatment was effective in eradicating BV in pregnancy with a risk ratio of 0.42 (32). A meta-analysis published recently evaluated the clinical cure rates (CCRs) for BV based on randomized controlled trials of different therapies and administration routes. The highest *P*-scores in CCRs were obtained by a combination therapy with probiotic treatment and the application of antibiotics (oral clindamycin and local 5-nitroimidazole) (33). However, a clear-cut decision for the best BV treatment was not possible due to the heterogeneity of outcomes reported in those trials (33). In our study, Fluomizin® was used to treat women with BV. It had been shown that vaginal Fluomizin® tablet was as effective as Clindamycin cream with a cure rate of 81.5% and 78.4% respectively (34). However, treatment was not given to two patients with BV due to imminent deliveries. The antenatal treatment impact on neonatal morbidity is still undefined. In our series, in the absence of therapy, there was a BV positive patient who presented with preterm labour at 32 weeks of gestation, but delivered soon after admission to a baby weighing 2,100 grams with a cord pH of 7.24. The baby required NICU admission, was intubated and was diagnosed to have RDS. The placental HPE confirmed acute chorioamnionitis. Another BV positive patient presented at 34 weeks of gestation and delivered within 5 h of presentation to a baby weighing 2,470 grams. The baby did not require NICU care and was discharged well to the mother without complication, although placental HPE was suggestive of acute chorioamnionitis.

Lastly, the isolation of GV in patients with BV infection had been reported by previous studies. It had been shown that a high GV load was associated with an increased risk of preterm birth and intra uterine growth restriction. Therefore, the role of GV in bacterial biofilm should not be overlooked (23, 35, 36, 37, 38). We managed to isolate GV in two-thirds of our patients with BV using the qualitative PCR technique. This was relatively lower as compared to a study by Spiegel et al. in which GV was isolated in all 25 patients diagnosed to have bacterial vaginosis (39); as well as a review by Aroutcheva et al. in which GV has isolated in 28 (87.5%) women with BV (40). This could be due to the difference in our population, in the assay method used. To date, there is no study comparing the performance of different methods of PCR assays in detecting GV. However, Caliendo et al. (41) in their study compared the performance of the qualitative and quantitative PCR assays for cytomegalovirus DNA in the plasma, having shown that qualitative assays had lower sensitivity than the quantitative ones.

The strength of our study is that we screened, diagnosed and treated BV early. It is the unit protocol to use Fluomizin rather than the Center of Disease Control recommendation of using Metronidazole 500 mg orally 2 times/day for 7 days OR Metronidazole gel 0.75% one full applicator (5 g) intravaginally, once a day for 5 days OR Clindamycin cream 2% one full applicator (5 g) intravaginally at bedtime for 7 days (42). Using Fluomizin was based on local availability and preference, although not robustly evidence-based. However, the use of a non-antibiotic agent may reduce the development of antimicrobial resistance. This study was not intended to compare treatments, as such future trials are needed to investigate this aspect. Nevertheless, we speculate that early treatment following diagnosis of BV in pregnant women with vaginal discharge, preterm labor or PPROM may have reduced the extent and limited the severity of preterm births, or perhaps adverse outcomes such as perinatal loss or mortality. We also investigated if babies were of lower birth weight when exposed to GV antenatally following our animal model that showed this outcome (38). Further studies are required to confirm this.

There were several limitations in this study. We only included symptomatic women that presented with abnormal vaginal discharge, preterm labour or PPROM. Thus, the prevalence of BV in this study might not represent the entire population. Larger population studies are needed to confirm the attributable risk for preterm birth in pregnant women with BV. We have also focused on GV as the sole aetiology of BV. The selection criteria could not tease out the different risk factors predisposing to BV and how these may affect outcomes separately as the numbers in the preterm labour and PPROM groups separately were small. Although our study showed that BV was associated with preterm births, we did not include information such as cervical length or fibronectin test as well. Future larger studies looking at preterm labour itself are needed to confirm that BV hastens delivery in such circumstances. The need for tocolytic treatment and the use of dexamethasone was individualized, which should have been controlled in future trials to prevent bias in the reported outcomes. More sophisticated bacterial detection and identification, and real-time PCR testing to relate the severity of bacterial load against disease and outcomes may be some considerations to incorporate in future trials.

## Conclusion

Our study showed that BV in pregnancy is associated with a significant risk of infants born preterm, with the interval from diagnosis of BV to delivery on average more than 2 weeks shorter than women without BV. Infants born preterm at a lower median birth weight are associated with an increased risk of neonatal morbidities such as RDS requiring NICU admission and respiratory support. There was no significant association with adverse maternal outcomes such as primary PPH and endometritis. More research is needed to study the preventive, diagnostic, and therapeutic approaches in association with a primarily adverse fetal outcomes as a result of BV in pregnancy.

## Data Availability

The raw data supporting the conclusions of this article will be made available by the authors, without undue reservation.
